# Marginal predation: do encounter or confusion effects explain the targeting of prey group edges?

**DOI:** 10.1093/beheco/arx090

**Published:** 2017-07-27

**Authors:** Callum Duffield, Christos C Ioannou

**Affiliations:** 1 School of Biological Sciences, University of Bristol, Bristol BS8 1TQ, UK

**Keywords:** aggregation, animal groups, edge effect, marginal predation, stickleback, virtual prey

## Abstract

Marginal predation, also known as the edge effect, occurs when aggregations of prey are preferentially targeted on their periphery by predators and has long been established in many taxa. Two main processes have been used to explain this phenomenon, the confusion effect and the encounter rate between predators and prey group edges. However, it is unknown at what size a prey group needs to be before marginal predation is detectable and to what extent each mechanism drives the effect. We conducted 2 experiments using groups of virtual prey being preyed upon by 3-spined sticklebacks (*Gasterosteus aculeatus*) to address these questions. In Experiment 1, we show that group sizes do not need to be large for marginal predation to occur, with this being detectable in groups of 16 or more. In Experiment 2, we find that encounter rate is a more likely explanation for marginal predation than the confusion effect in this system. We find that while confusion does affect predatory behaviors (whether or not predators make an attack), it does not affect marginal predation. Our results suggest that marginal predation is a more common phenomenon than originally thought as it also applies to relatively small groups. Similarly, as marginal predation does not need the confusion effect to occur, it may occur in a wider range of predator–prey species pairings, for example those where the predators search for prey using nonvisual sensory modalities.

## Lay Summary

Living in groups has been shown to have antipredator benefits for prey animals but risk still differs for individuals within a group. Using fish as predators presented with simulations of virtual prey, we show that prey on the edge of groups are more likely to be attacked than others, even at relatively small group sizes. We also show that encountering prey on the edge is likely to explain this effect rather than predatory confusion.

## INTRODUCTION

Individuals that form groups gain many fitness advantages, with the most widespread being a reduced risk of predation (Krause and Ruxton 2002; Ioannou et al. 2011; Santos et al. 2016; Ward and Webster 2016). Within a group, the risk of predation is rarely homogenous due to phenotypic variation in size, speed, age, sex, and color (including warning, aposematic coloration), among other factors (Ohguchi 1978; Theodorakis 1989; Riipi et al. 2001; Rodgers et al. 2014). Even within groups of prey that are very similar in phenotype, however, individuals will still vary in their spatial positions. Numerous theoretical and empirical studies have demonstrated that individuals on or closer to the margins (edges) of a group have a much higher risk of predation than those found nearer the center (Hamilton 1971; Vine 1971; Krause 1994; Bumann et al. 1997; Viscido et al. 2001; Ballerini et al. 2008; Morrell and James 2008; Morrell and Romey 2008; Hirsch and Morrell 2011), a phenomenon known as marginal predation or the edge effect. Nevertheless, the mechanisms behind this higher rate of marginal attack are still debated (Krause and Ruxton 2002; Caro 2005; Quinn and Cresswell 2006). The 2 explanations that are most commonly discussed are the confusion effect and the greater likelihood of encountering individuals on the group’s edge.

The simpler, and therefore more widely applicable, of the 2 mechanisms is based on the encounter rate between a predator and the individuals in a prey group. This assumes that a predator will attack the closest prey to them (Hamilton 1971; Vine 1971; Hirsch and Morrell 2011), which is most commonly prey on the margins of groups, thus explaining the high rate of marginal predation. Due to its simplicity, encounter rates provide a very general mechanism and has been an assumption of predator–prey behavioral models (Wood and Ackland 2007). The second major explanation, the confusion effect (Neill and Cullen 1974; Miller 1992; Ioannou et al. 2008; Scott-Samuel et al. 2015), relies on the cognitive limitations of predators. The confusion effect assumes that prey in larger or more dense groups are more difficult to track and attack compared to lone prey due to the larger amount of information in the visual field of the predator from more possible targets or prey overlap (Krakauer 1995; Lemasson et al. 2016). If the information presented leads to an overload of the predator’s cognitive ability, this can result in a lower rate of attacks (Ioannou et al. 2009), lower attack success (Krause and Godin 1995; Ioannou et al. 2008), and a reduced cognitive capacity for other activities such a vigilance (Milinski and Heller 1978; Milinski 1984). These costs, if high enough, present predators with the choice of not making an attack or finding ways to reduce the confusion effect. One way to reduce this confusion is to focus attacks on the periphery of the group, which can have the effect of reducing the number of prey in the visual field and hence the strength of the confusion effect (Ioannou et al. 2009; Rieucau et al. 2014).

The confusion and encounter explanations for marginal predation are not mutually exclusive, but are driven by fundamentally different aspects of predator behavior. Assuming a prey individual is chosen at random, the probability of targeting an individual on the edge of a group is dependent on the ratio of peripheral versus central individuals, so if groups become denser but the total area occupied by the group is constant, then predators should not be more likely to attack marginal prey as the ratio of peripheral to central individuals remains constant. In contrast, an increase in prey density induces a stronger confusion effect (Milinski 1979, 1984; Ioannou et al. 2009; Scott-Samuel et al. 2015) due to the cognitive limitation of attacking prey when many other targets are visible. Thus, if predator confusion is the driving mechanism, then the tendency to attack marginal prey should increase with the density of prey even if the ratio of peripheral to central individuals is constant.

While both encounter and confusion effects assume that groups contain phenotypically similar individuals, this is difficult to replicate with live prey as there are many sources of interindividual variation that influence predator attack rates including prey size, color, and activity (Wetterer 1989; Bell and Sih 2007; Ioannou and Krause 2009; Hogan et al. 2016). To negate this problem, virtual prey simulations are being increasingly used for predator–prey experiments (Lemasson et al. 2016). Virtual prey are used in both cognitive psychology and animal behavior due to the practical advantages that it provides (Bond and Kamil 2002; Harland and Jackson 2002; Ioannou et al. 2012; Lemasson et al. 2016; Hogan et al. 2017). Virtual prey not only allow for the standardization of individual phenotypes but also enables the experimenter to control other factors such as prey behavior, number and density without unintended confounding effects, and the ethical issues in using live animals as prey.

Previous studies have used large group sizes to assess marginal predation, from group sizes of 20 individuals (Romey et al. 2008) to as large as 100 (Hirsch and Morrell 2011). These studies have shown that both encounter rate and the confusion effect are credible explanations of marginal predation; however, in nature, group size distributions are often heavy-tailed, with many smaller groups and relatively few larger groups (Krause and Ruxton 2002). To fully understand the extent of marginal predation, it is important to determine how predators prey upon smaller prey group sizes. Yet to date there have been no studies investigating how large a group needs to be before marginal predation is observed, and the relative importance of encounter and confusion effects in explaining marginal predation. Here we quantify predator behavior when presented with manipulated groups of virtual prey (Ruxton et al. 2007; Jones et al. 2011), using 3-spine sticklebacks as predators. We test the minimum group size required for marginal predation to be observed by varying the number of individuals within a group. We then test the mechanism(s) behind marginal predation by altering group density. By varying the density and keeping area constant, the proportion of individuals on the edge of the group and the average distance of prey from the center remains equal and so should not affect encounter rate. However, as density increases, the amount of prey presented to predators increases, and so higher densities should induce more confusion in the predators.

## METHODS

### Subjects and housing

Approximately 300 three-spined sticklebacks (*Gasterosteus aculeatus*) were caught on 25 September 2014 from the River Carey, Somerset, UK (ST 469 303), using keep nets dragged though vegetation. Once at the lab, fish were kept in 40 × 70 × 34 cm (width × length × height) glass tanks on a flow through system. Water temperature was maintained at 16 °C with a daily 12:12 dark:light cycle throughout the study, which maintained the nonbreeding condition of the fish. When not taking part in experiments, the fish were fed defrosted bloodworms and tropical flake food. Upon completion of the experimental tests, all fish were kept in the lab for further behavioral experiments. All necessary permits were obtained to remove fish from the wild and hold them at the University of Bristol, and all experimental procedures were within the guidelines set by the University of Bristol and the Association for the Study of Animal Behaviour.

### Experimental methods and apparatus

**Figure 1:**
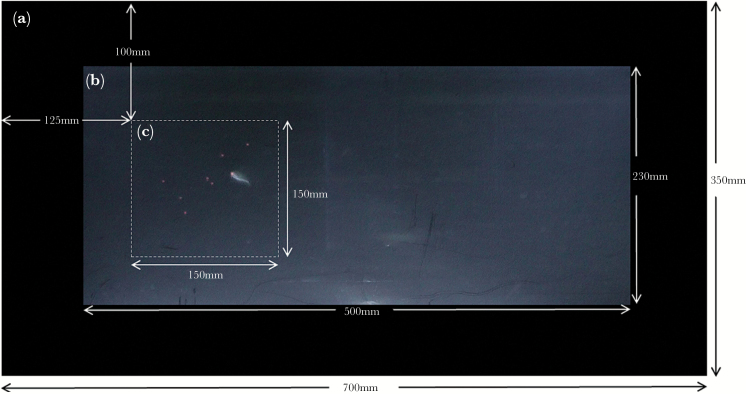
The front view of the experimental tank showing the black corrugated plastic screen border (a), the translucent screen and field of view of the camcorder (b), and the projected prey arena (c) containing a stickleback attacking one of 8 prey.

To assess the predatory behaviors of sticklebacks when attacking groups of prey, we conducted 2 experiments that altered either the number of prey and the area they occupied (Experiment 1: group size) or the number and density of prey but kept the area they occupied constant (Experiment 2: group density). In both experiments, an agent-based model was used to simulate groups of 2D circular red prey, adapted from Wilensky (1999) and run in Netlogo 5.1.0. In both Experiments 1 and 2, the projected prey (agents) had a diameter of 2.5 mm, moving at a constant speed of 0.02 cm/s. Each trial had a unique random seed which determined initial positions and orientations and hence determined the paths prey took. In both experiments, the prey types followed 2 basic rules: separate and cohere. “Separate” kept agents a minimum distance apart and “cohere” made agents aggregate. Parameters were set to consistently give a single group of prey in a swarm-like state (Couzin et al. 2002). In Experiment 2, to manipulate prey density, prey groups consisted of visible (red) and nonvisible (transparent) prey totaling 32 individuals (Lemasson et al. [2016] have recently used a similar technique when investigating the confusion effect in humans). By altering the number of visible to nonvisible prey, density can be manipulated while the area occupied by the group’s circumference remains constant. The simulation was projected on to a translucent screen (Rosco gel No. 252) that was affixed to the inside of the experimental tank (Supplementary Figure S1). The projector (BenQ MW523) was positioned approximately 100 cm in front of the tank and 50 cm below the tank, such that the projector was at an angle to reduce glare on the front of the tank. The simulated arena measured 15 × 15 cm, with the top left corner positioned 10 cm from the top of the tank and 12.5 cm from the left edge of the tank wall that the prey were projected onto (Figure 1).

Experiment 1 (group size) used different group sizes of 2, 4, 8, 16, or 32 agents with the same interindividual spacing so that the prey groups varied in group size and area, but not in density. The periphery to area ratio thus decreased as group size increased, that is there were fewer prey on the periphery compared to the center of the group. In Experiment 2, the group density treatments varied group size but rather than keeping density (prey per unit area) constant and allowing the area taken by the group to vary (as in Experiment 1), we manipulated the ratio of visible to nonvisible prey (32:0, 24:8, 16:16, 8:24). This meant that all prey density treatments took up the same area (with the same periphery: area ratio) and the density and number of (visible) agents varied.

For Experiment 1, on each test day at 13:00, 5 “companion” fish were placed in each of the 2 companion compartments (Supplementary Figure S1) and were used until all trials had been conducted that day. For Experiment 2, companion fish were placed in the compartments at the start of the day until 13:00 at which point they were replaced with another 10 fish which were then used for the remainder of the day. The companion fish were used to habituate the fish to its new environment and to promote the perception of food competition (Grand and Dill 1999), both of which are expected to motive the test fish to attack the virtual prey. Prey treatments within each experiment were ordered in a complete random block, with each treatment appearing once in each block but in a random order. In total, 29 trials were conducted for group size treatments of 2, 8, and 16; 30 trials were conducted for group size treatments of 4 and 32; and 46 trials were conducted for each density treatment. At the start of each trial, the simulation was set up with a unique randomly generated seed (ranging between 1 and 10000) and was allowed to run for at least 500 “ticks” (i.e., time steps) before a fish was placed into the experimental tank. We used a net to transfer an individual from the holding tanks to the refuge in the experimental tank, and once the fish was placed and the net removed, the trial began. The trial was terminated once the individual had made an attack or 600 s had elapsed from starting the trial. The individual’s standard body length was then measured to the nearest millimeter (ranging between 21 and 43 mm, mean = 30.80 mm) and the fish placed in a different holding tank before all fish were returned to their main tank to avoid using the same fish more than once within each experiment. Experiments were recorded 108 cm from the front of the tank using a Panasonic SD800 camcorder and also from above using a webcam positioned 34 cm above the water. The webcam was used to monitor movements of the test fish during the experiment to establish when they left the refuge (whole body being outside of area C; Supplementary Figure S1). The camcorder footage was used to measure the latency to the first attack, which individual agent was attacked and how many attempts were made in their first bout of attacks (fish were not limited in their attempts but were limited to one bout before the trial was ended [Ioannou et al. 2008]). A total of 147 trials were conducted in Experiment 1 (always in the afternoon between 13:00 and 18:00) and 230 trials were run in Experiment 2 (between 09:00 and 18:00), with each trial using a different fish within an experiment but the same fish being used between experiments. To motivate fish to make an attack and to standardize hunger, fish were not fed the previous day but were fed upon being returned to their main tank after all trials were completed for that day.

### Statistical analysis

As the positions of prey relative to one another varied stochastically as the simulation ran, the prey group stimulus at the time of attack differed between trials. We thus analyzed prey traits using randomization tests to compare the observed frequencies of attacking particular prey traits to those expected if each fish selected a prey randomly from the same set of prey stimuli (see Ioannou and Krause [2009] and Ioannou et al. [2012] for a similar approach). Specifically, these traits were: whether prey were on the edge of a group, whether their distance from the center of the group was greater than the mean value, and the side of the group they were on (near or far side relative to the refuge the fish started from). For each iteration, an individual prey was randomly selected from the prey at the point of the first attack in each trial where an attack occurred. This was repeated for 10000 iterations. This yielded expected distributions for the number of trials where prey were attacked on the edge, whether they were closer to the group center than average, and whether they were attacked on the near or far side of the group. If the observed number of trials where the targeted prey had each trait was <2.5% or >97.5% of this expected random-targeting distribution (i.e., α = 0.05), the observed attacking behavior could be said to be statistically different to targeting prey at random. These tests were carried out separately for each treatment in each experiment.

To define whether a prey was on the edge of the group, we used the deldir package in R v3.1.2 to calculate each individual’s domain of danger using Voronoi tessellation (Hamilton 1971). Any individual prey that had a Voronoi cell not entirely bounded by the Voronoi cells of other individuals was classed as being on the edge of the group, that is, had the boundary of the simulated space as part of its Voronoi cell. To determine the side of the group individuals attacked, we determined whether the prey was on the near or far side from the refuge based on their position relative to the group centroid. For determining distance from the center, we calculated the group centroid and then the mean distance from the center for all prey in the group. We then determined which individuals had distances to the center greater than the group mean. This has been a measure of marginal predation in previous studies (Romey et al. 2008; Hirsch and Morrell 2011) and so we use this measure here too. However, this measure does not necessarily represent marginal predation since prey slightly further from the mean distance from the centroid may still be far from the margins of the group and thus be as confusing as “central” prey.

To analyze factors affecting average levels of predator behavior, we used Generalized Linear Models (GLMs). All models contained 2-way interactions except for time of day which was not included in any interactions. To find the minimum adequate model, the dropterm function from the package MASS was used to identify the least significant terms, which were sequentially removed from the model until only significant terms remained. Tests were carried out separately for Experiments 1 and 2. When models were dependant on an attack occurring (i.e., side of the prey group an attack was made, time to make an attack, number of attacks made in the first bout, whether an attack was on the edge of the group, the relative distances of attacked prey from the mean distance to the centroid, and whether an attack was further than the mean distance to the centroid), only trials where an attack was made were included.

We used GLMs with negative binomial distributions and log-link functions to determine factors influencing the time taken to leave the refuge, the time taken to make an attack, and the number of attacks made. Prey group treatment and subject body size were used as fixed effects for analyzing the time out of the refuge. Prey group treatment, body size, and time out of the refuge were used as fixed effects for analyzing the time to make an attack. Prey treatment, body size, time out of the refuge, and time to make an attack were used as fixed effects for analyzing the number of attacks made.

A GLM with a binomial distribution and logit-link function was used to determine the factors influencing whether each fish made an attack. Due to the variable times taken to leave the refuge and the fixed total trial time of 600 s, the time available for the fish to make an attack (once leaving the refuge) varied between trials. Thus, to analyze the probability of making an attack, we excluded all trials where the fish took more than 360 s to make the attack. A threshold of 360 s allowed us to standardize the time available and minimized the number of trials excluded (Experiment 1 [group size]: 23 trials out of 147 were excluded; Experiment 2 [group density]: 17 trials out of 230 were excluded). Prey treatment, body size, and time to leave the refuge were used as fixed effects.

We also used GLMs with a binomial distribution and logit-link function to determine the factors influencing the side of the group the fish attacked (i.e., near or far side relative to the refuge), whether an attack was on the edge of the group, and whether an attack was further than the mean distance to the centroid. Prey treatment, time out of the refuge, time to make an attack, and body size were used as fixed effects. In Experiment 1, the group size 2 treatment was excluded from GLMs assessing whether an attack was made further from the centroid than the mean prey distance and when an attack was made on the edge of the group based on Voronoi tessellation since the 2 prey would be the same distance from the center and always on the edge.

All statistical analyses were performed in R version 3.1.2 (R Core Team 2014). The GLMs with binomial and negative binomial distributions met their assumptions and were not over or under dispersed (i.e., the dispersion parameter was between 0.5 and 2.0).

## RESULTS

### Experiment 1: group size

**Figure 2:**
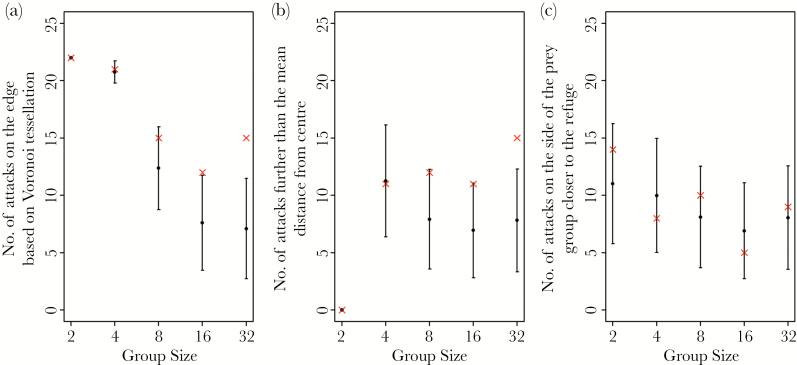
The number of times for each group size that the observed attacks (cross) and random simulation of 10000 iterations (filled circles are means, with error bars showing 95% confidence intervals) was (a) on the edge, (b) further from the group centroid than the mean, and (c) to the far side of the group center relative to the refuge.

Sticklebacks were significantly more likely to attack prey on the edges of groups (Figure 2a) and also further from the group center (Figure 2b) compared to simulations where prey were selected randomly. This effect became statistically significant in relatively small groups of 16 or more prey (prey on the edge, group size 16: *P* = 0.016, group size 32: *P* < 0.001; distance to center, group size 16: *P* = 0.023, group size 32: *P* < 0.001). When comparing which side of the group was attacked (near or far from the refuge), there was no difference between the observed results and the randomized simulation for any group size treatment (*P* ≥ 0.138 in all treatments, Figure 2c).

**Figure 3:**
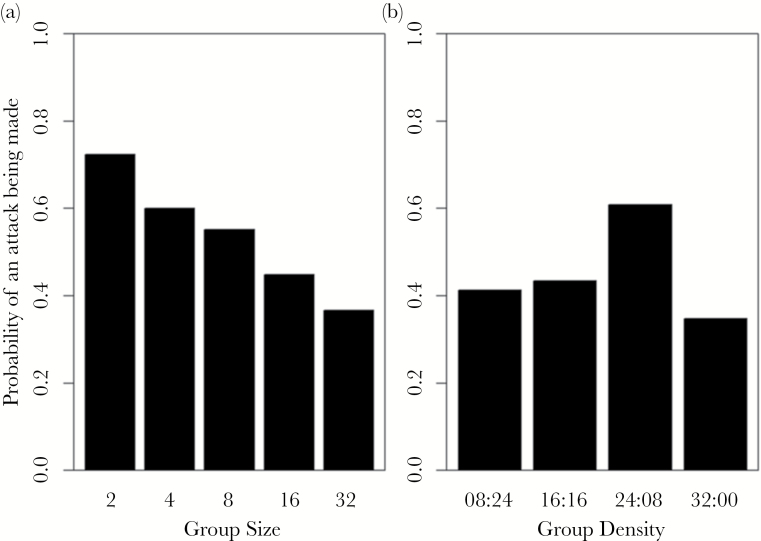
The probability of a stickleback attacking virtual prey within 360 s at different prey (a) group sizes (Experiment 1) and (b) group densities (Experiment 2).

The likelihood of whether an attack was made was significantly affected by group size (GLM: LRT_1,146_ = 8.40, *P* = 0.004, Figure 3a), with attacks being less likely as group size increased. The body length of the subject also had a significant effect on the likelihood of an attack (LRT_1,146_ = 4.34, *P* = 0.037), with smaller individuals being more likely to make an attack than larger individuals. It was also found that individuals tested earlier in the day were significantly more likely to make an attack than individuals tested later in the day (LRT_1,146_ = 5.22, *P* = 0.022). The time taken for the individual to leave the refuge had no significant effect (LRT_1,144_ = 0.23, *P* = 0.635).

**Figure 4:**
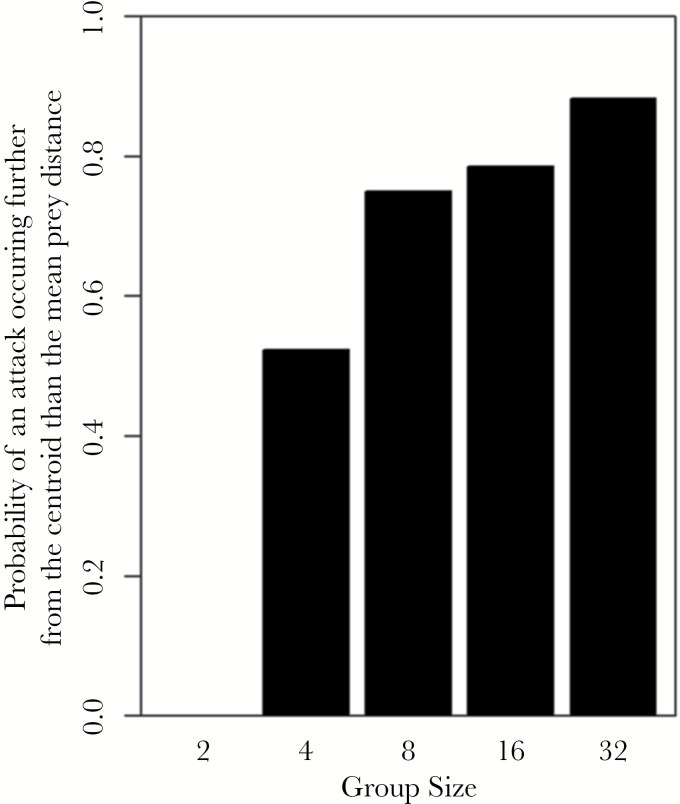
The probability of an attack being made further from the centroid than the mean distance of prey to the centroid as a function of prey group size (Experiment 1).

When an attack was made, we found that edge attacks, based on Voronoi tessellation, were not significantly affected by group size (GLM: LRT_1,88_ = 1.69, *P* = 0.194) or any other factors. However, we found that group size had a significant effect on whether an attack was made further from the centroid than the mean prey in the group (GLM: LRT_1,89_ = 5.44, *P* = 0.020, Figure 4). The probability of attacking prey further from the centroid than the mean prey distance from the centroid increased as group size increased. When testing factors influencing whether attacks were targeted at prey on the side of the group relative to the refuge, group size had no significant effect (GLM: LRT_1,89_ = 0.25, *P* = 0.614). All other factors were also nonsignificant. The time taken to leave the refuge, time taken to make an attack, and the number of attacks were all found not to be significantly affected by any of the factors tested (*P* > 0.05 in all cases).

### Experiment 2: group density

**Figure 5:**
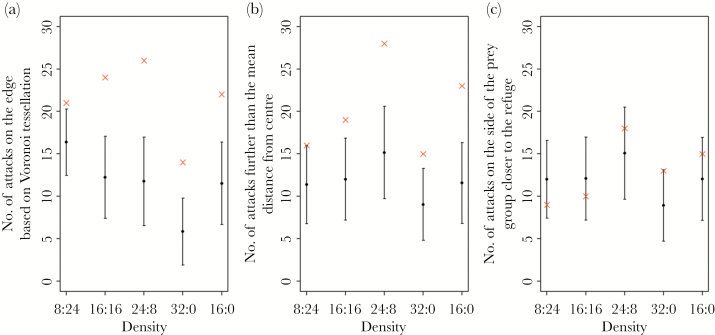
The number of times per prey density that the observed attacks (cross) and random simulation of 10000 iterations (filled circles are means, with error bars showing 95% confidence intervals) was (a) on the edge, (b) further from the group centroid than the mean, and (c) to the far side of the group center relative to the refuge.

Sticklebacks were significantly more likely to attack prey on the edge of groups (Figure 5a) and further from the group centroid (Figure 5b) compared to the simulations where prey were selected randomly. This was statistically significant across all density treatments for prey on the edge (*P* ≤ 0.011 in all treatments) and all density treatments for distance from the center except 8:24 (*P* ≤ 0.004 in all treatments except 8:24 where *P* = 0.034). As in the first experiment, the observed side of the group that was attacked relative to the refuge was found not to be significantly different to the simulation for any density treatments (*P* ≥ 0.045 in all treatments, Figure 5c; note that the 2-tailed alpha in these randomizations is 0.025).

**Figure 6:**
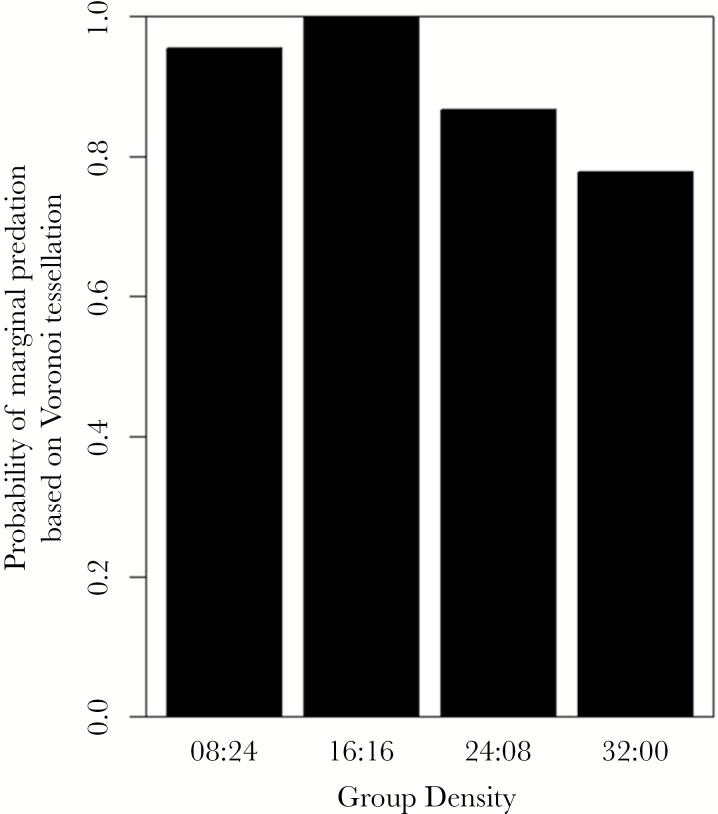
The probability of an attack being made on the edge of a group as described by Voronoi tessellation at different prey group densities (Experiment 2).

Prey density (Figure 3b), fish body size, time taken to leave the refuge, and time of day were all found to have nonsignificant effects on whether an attack was made (*P* ≥ 0.07 in all cases). However, when an attack was made, density was found to have a significant effect on whether an attack was made on the edge of the prey group based on their Voronoi cells (GLM: LRT_3,93_ = 8.57, *P* = 0.036). Sticklebacks were less likely to attack the edge of a prey group if the prey groups were more dense (Figure 6). We also found that the interaction between prey group density and the time to leave the refuge had a significant effect on whether the prey individual attacked was further from the centroid than the mean prey distance (GLM: LRT_3,93_ = 16.36, *P* < 0.001, Supplementary Figure S2). We found that attacks were increasingly likely to be made further from the centroid than the mean prey when fish took longer to emerge from the refuge and were presented with the least dense group (08:24) and the densest group (32:00). However, the group densities of 16:16 and 24:08 had a high probability of making an attack on prey further from the centroid regardless of how long sticklebacks took to emerge from the refuge. The interaction of prey density and fish body size also had a significant effect on whether prey was attacked further from the centroid than the mean prey distance (LRT_3,93_ = 7.82, *P* = 0.050). In all density treatments except 24:08, it was found that the probability of an attack occurring further from the center decreased as body size increased; in other words, larger fish were more likely to attack central individuals. In contrast, the 24:08 treatment showed that as body size increased, the probability of an attack occurring further from the centroid than the mean prey distance also increased.

The side of the prey group that was attacked was shown to be significantly affected by the interaction between the time out of the refuge and prey density (GLM: LRT_3_ = 11.90, *P* = 0.008, Supplementary Figure S3). Both the densest and the least dense prey group were more likely to be attacked closer to the refuge if sticklebacks left the refuge quickly, and attacks closer to the refuge were very unlikely after 300 s. However, the reverse was observed on densities of 16:16 with a smaller probability of attack occurring close to the refuge when sticklebacks emerged from the refuge earlier.

**Figure 7:**
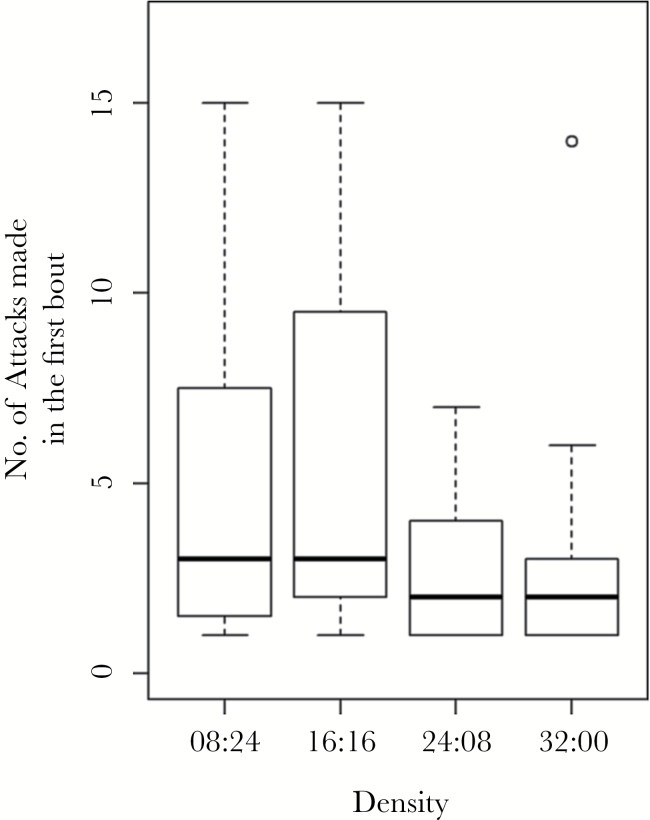
The number of attacks made in the first bout on prey groups of different densities (Experiment 2). The thick black lines represent the medians, the boxes encompass the 25th–75th percentiles and the most extreme points within ±1.5× the interquartile range outside the box are denoted by the whiskers. The circles show points lying outside these ranges.

The time taken to leave the refuge was significantly affected by body size (GLM: LRT_1,183_ = 7.37, *P* = 0.007), with larger individuals being more likely to leave the refuge sooner. However, the time taken to make an attack was not significantly affected by any factors tested for here. Prey density was shown to have a significant effect on the number of attacks made in the first attack bout (GLM: LRT_3_ = 10.40, *P* = 0.016, Figure 7). Individuals that were presented with denser prey groups made less attacks than those that were presented with less dense groups. The number of attacks was also significantly affected by the body size of individuals (LRT_1_ = 6.10, *P* = 0.014), with larger individuals making more attacks than their smaller counterparts (Tables 1 and 2).

**Table 1:** Summary of results for the simulation vs. observed data for both group size and group density trials

Response variable	Group size simulations				Group density simulations			
	Group size	*N*	No. of iterations *N* ≤ simulation	*P* value	Group density	*N*	No. of iterations *N* ≤ simulation	*P* value
No. of attacks made on the edge	2	22	10000	1.000	08:24	21	106	0.011*
	4	21	7502	0.750	16:16	24	0	<0.001***
	8	15	827	0.083	24:08	26	0	<0.001***
	16	12	157	0.016*	32:00	14	1	<0.001***
	32	15	0	<0.001***	16:00	22	0	<0.001***
No. of attacks further from the center than the mean prey distance	2	0	10000	1.000	08:24	16	344	0.034
	4	11	6336	0.634	16:16	19	25	0.003**
	8	12	320	0.032	24:08	28	0	<0.001***
	16	11	230	0.023*	32:00	25	33	0.003**
	32	15	1	<0.001***	16:00	23	0	<0.001***
No. of attacks made on the prey group side closest to the refuge	2	14	1382	0.138	08:24	9	9415	0.942
	4	8	8723	0.872	16:16	10	8600	0.860
	8	10	2459	0.246	24:08	18	1935	0.194
	16	5	9063	0.906	32:00	13	440	0.044
	32	9	4079	0.408	16:00	15	1571	0.157

*N*, the number of prey arrays used for the random simulation, taken from trials whereby an attack was made upon the virtual prey array by a stickleback.

*Significant at *P* < 0.025.

**Significant at *P* < 0.005.

***Significant at *P* < 0.001.

**Table 2:** Summary of significant results from the GLMs conducted

Response variable	Explanatory variable	d.f.	LRT	*P* (Chi)
Significant group size GLMs				
Probability of an attack being made	Group size	1	8.40	0.004**
	Body size	1	4.34	0.037*
	Time of day	1	5.22	0.022*
Probability of the attacked prey being further from the center than the mean prey distance	Group size	1	5.44	0.020*
Significant group density GLMs				
Time to leave the refuge	Body size	1	7.37	0.007*
Probability of the attacked prey being further from the center than the mean prey distance	Group density: time to leave refuge Group density: body size	33	16.367.82	<0.001***0.050*
Probability of an edge attack	Group density	3	8.57	0.040*
Probability of the side closest to the refuge being attacked	Time to leave refuge: group density	3	11.90	0.008*
No. of attacks made in the first bout	Group density	3	10.49	0.015*
	Body size	1	6.37	0.012*

GLM, Generalized Linear Models.

*Significant at *P* < 0.025.

**Significant at *P* < 0.005.

***Significant at *P* < 0.001.

## DISCUSSION

Confusion and encounter effects have both been argued as key mechanisms driving marginal predation (Hamilton 1971; Vine 1971; Milinski 1977a,b). Research on this topic has, however, mostly focused on large group sizes (Milinski 1977b, 1979; Romey et al. 2008; Hirsch and Morrell 2011), yet in nature much smaller group sizes can be observed (Krause and Ruxton 2002). Previous studies have also favored either the confusion or encounter mechanisms rather than considering both. Here, we show that marginal predation can be seen at group sizes as small as between 8 and 16, and while there was evidence for predator confusion in our experimental system, it does not appear to be the main mechanism driving marginal predation.

In Experiment 1, sticklebacks were more likely to attack the edges of a prey group compared to the random simulation when the group size was 16 or greater, and in Experiment 2, we found that edge attacks were more likely to occur in all treatments, including in a group of 8 where the number of attacks on the edge did not differ from random in Experiment 1 (although the number of attacks, and hence test power, was greater for 8 visible prey in Experiment 2). This shows that group sizes do not need to be particularly large for marginal predation to occur, and that prey density can be low and still induce marginal predation. If confusion is the main driver of marginal predation, then it would be expected that with an increase in confusion, the marginal predation effect would become more prominent (Krakauer 1995). However, the effect of group size was not significant when examining its effect on edge attacks. Due to the nature of Experiment 1, as group size increases, the group periphery (i.e., edge): area ratio decreases so there are relatively fewer prey on the periphery of larger groups, which would counteract an increased preference for attacking group margins in larger groups. Thus being able to draw conclusions from edge attacks is difficult. To negate this, in Experiment 2, this ratio was kept constant allowing for direct comparison between different group treatments. Nevertheless, the effect of density on marginal predation had a significant effect in the opposite trend to what would be expected from the confusion effect, where attacks on denser groups were less likely to be made on marginal prey. This suggests that confusion is not the main driver of marginal predation in our system. However, this result also does not support the encounter mechanism, which would predict density to have no effect on marginal predation.

If encounter was the only mechanism acting on marginal predation then it would be expected that in all treatments, attacks would also be more likely to be made on the side of the group closest to the refuge. Yet, this was not seen to be significantly different in either experiment when compared to the randomized simulation. This shows that sticklebacks do not just attack the closest prey to them, but factors such as the possibility of predation and competition may be causing our predators to explore the arena before they make an attack. At one spatial scale (from leaving the refuge), an encounter effect does not seem to occur, while at a smaller scale, prey on the edges of groups do seem to be preferentially attacked once the predator is close to the group. While the importance of scale has been recognized in ecology for some time (e.g., Gunton and Kunin 2007), it has been neglected in behavioral predator–prey studies (Ioannou et al. 2009).

A metric commonly employed to quantify the effects of confusion on marginal predation is the distance from the centroid of the group. In Experiment 1, we found that prey were more likely to be attacked further from the centroid than the mean prey distance. However, while it has been previously shown that distance from centroid and predation risk correlate (Romey et al. 2008; Hirsch and Morrell 2011), this may not always be indicative of a confusion effect. The confusion effect explanation for predators attacking the prey group edge is an attempt to reduce confusion due to prey having a smaller number of neighbors. Yet prey further from the center than the mean could potentially be just as confusing as other central prey, especially in larger groups.

This lack of a confusion effect may be a consequence of the predator–prey system we used, with our prey group being relatively stationary, exhibiting no antipredatory responses and being constrained to a 2D plane. If prey groups were more mobile, as is commonly observed (Mills and Shenk 1992; Hebblewhite and Merrill 2007), predatory confusion could increase. If prey groups remain stationary (Gross and MacMillan 1981), a predator can get much closer and select a smaller subgroup before having to process information since the group will likely still be in the same area. This could also be the case for prey that exhibit evasive maneuvers or increased aggregation (Hamilton 1971; Treisman 1975; Lemasson et al. 2016). Again, if prey made decisions based on the predator’s movements, predators will have to constantly update their information, potentially increasing the effects of confusion (Walther 1969; Treherne and Foster 1980; Jakobsen and Johnsen 1988). Prey could also be in a 3D plane as opposed to our 2D prey system which could influence the confusion effect. Even though predators attacking a 2D prey group from a 3D plane can be observed in the wild (Treherne and Foster 1980; Foster and Treherne 1981), predator–prey systems occurring within the same plane (such as lions attacking heard of ungulates: Madsen and Shine 1996, or birds of prey on bird flocks: Quinn and Cresswell 2006) could increase the effects of confusion. When predator and prey groups share the same plane, the prey groups will exhibit more prey overlap (from the predators’ perspective) and prey group edges would be more difficult to establish (Romey et al. 2008; Hogan et al. 2017).

Nevertheless, our study was intentionally based on a simple, relatively stationary prey system since we aimed to tease apart the mechanisms behind marginal predation. Yet, in the natural world, group dynamics vary between species with interactions of group size and density occurring, uneven densities (such as patchy or graduated density in nesting birds: Lahti 2001), or the whole group moving such as in migratory species (Madsen and Shine 1996). Individuals within the group also vary phenotypically (size, color, and sex among others) and behaviorally, varying in speed, amount of overlapping, and degree of turning or exhibiting antipredatory responses such as evasion and aggregation. These aspects of prey behaviors and variation will undoubtedly contribute to both encounter rates and confusion, but now that a flexible experimental approach has been established, further parameters can be added to assess their consequences. The basis of this study is therefore applicable to many other species and with the addition or variation of parameters, the causes of different predatory strategies such as marginal predation may become more evident. To further understand the effects of prey movements on predatory behaviors, future studies could also track predators as they approach a prey group. Unfortunately, this was not possible in our experimental setup but would provide further insight into the effects prey aggregations on predators. Nevertheless, it would be difficult to analyze predator trajectories to try and separate the mechanisms behind marginal predation as we try to do here. At some point, the attacked prey will always be the closest prey item and it would not be clear at what point the predator had chosen the individual, when it was the closest prey item (suggestive of encounter) or earlier to minimize confusion.

While confusion may not be driving marginal predation, at least in our experiment, other behaviors have been associated with predatory confusion, such as the probability of making an attack (Milinski 1984; Ioannou et al. 2008), the time to make an attack, and the number of attacks made in the first bout (Milinski 1977b). In Experiment 1, it was shown that less than 40% of individuals made an attack on larger groups compared with over 70% of individuals that made an attack on smaller groups, suggesting that confusion is an important mechanism in predatory behaviors (Milinski 1984). A similar result was found by Ioannou et al. (2008) where the number of attacks significantly decreased with a higher number and density of prey. Likewise, we found that as density of prey increased, the number of attacks made decreased (Milinski 1977b), although we did not find this in the first experiment. This further suggests that confusion is important in predator behavior in our experiments, with predators choosing not to attack more confusing groups or reducing the number of attacks made in a bout if an attack was initiated.

The number of attacks was also affected by the body length of an individual, with larger individuals making more attempts regardless of density. This suggests that individual variation is also important in predatory behaviors of sticklebacks (Hirsch and Morrell 2011). Since all prey in our study were of the same size, the size ratio of predator to prey would vary depending on the size of the fish (ranging between 5.81% and 11.90% of body length). This may have caused smaller fish to make less attacks simply due to the particular size of the prey, either since they would require fewer prey items to satiate or because the prey items were much bigger than they would usually attack (Turesson et al. 2002; Gill 2003). Yet, since the fish never gained a food reward for attacking the prey, they would not be likely to make fewer attacks due to being smaller, and so satiated sooner. It would also be expected that if prey items were too large, individuals would be more likely not to make an attack than to make an attack but with fewer attempts. It has been observed in several fish species that prey items between 10% and 20% of their body size are often taken suggesting that the prey items were unlikely to be too large for the sticklebacks in this study, even for the smallest fish (Scharf et al. 2000).

Furthermore, individual variation in body size was also seen to influence whether an attack was made further from the centroid than the mean distance. Experiment 2 showed that density in an interaction with body size had a significant effect on the distance from the centroid of attacked prey, with smaller individuals having a higher probability of attacking further from the centroid when presented with the densest treatment. For the least dense group, there was no effect of the size of the fish. This further shows that there may be an aspect of phenotypic variation in predators that influences their behavior (Milinski 1979). The time spent in the refuge also showed size-dependant variation with smaller individuals spending longer in the refuge than larger individuals. This is contrary to most other findings as larger individuals usually spend longer in the refuge, with hunger driving smaller individuals to leave the refuge sooner (Krause et al. 1998; Dowling and Godin 2002). However, Krause et al. (2000) showed that when in groups, larger individuals emerged from the refuge sooner than when alone, emerging as quickly as smaller individuals. They suggested that when foraging animals aggregate, the effect of competition between large individuals increases more than between smaller individuals. Our use of companion fish was designed to promote the perception that the test fish was in a group and we also deprived subjects of food for 24 h with food used as a stimulus, neither of which were used in Krause et al. (2000). This lack of food and apparent availability of food during the experimental tests could have driven competition in larger individuals more than usual, thus our results are consistent with the results of Krause et al. (2000). This clearly has implications for any species that form groups, indicating that individual size and internal state could influence the behaviors that they exhibit.

Our findings show that marginal predation is not restricted to large groups of prey but can be seen in groups at least as small as 16 individuals. While both explanations are relevant to predatory behaviors, we have shown that the confusion effect and encounter rate are driving different aspects of predator–prey behavioral interactions. We show that in small groups, attacks are more likely to be made which suggests that when confusion is too great, predators choose not to make an attack. Yet when attacks are made, it is encounter rates that are more likely to be explaining marginal predation. While encounter and confusion are both valid mechanisms explaining predatory behaviors, they operate at different stages of a predation event. Encounter rates are a more ecologically based mechanism, whereas confusion is more localized to an individual’s field of vision which may only be a subset of a group. We suggest that to advance our understanding of marginal predation, not only should smaller groups be considered, but also mechanisms be studied in parallel and with equal weight rather than focusing on one.

## SUPPLEMENTARY MATERIAL

Supplementary data are available at *Behavioral Ecology* Online.

## FUNDING

This work was supported by a Natural Environment Research Council Independent Research Fellowship (NE/K009370/1) to CCI.

## Supplementary Material

Supplementary Material

Duffield Figure S1

Duffield Figure S2

Duffield Figure S3
