# Supplementary material for: Marginal predation: do encounter or confusion effects explain the targeting of prey group edges?
Source: Behav Ecol. 2017 Jul 27;28(5):1283–92. doi: 10.1093/beheco/arx090 (PMC5873256; doi:10.1093/beheco/arx090)
Supplement: Supplementary Material [file arx090_suppl_supplementary_material.docx]

**Supplementary Material**

**Fig S1.** Experimental design of stickleback predator and virtual prey experiments showing the companion compartments (A and B), the stickleback refuge (C) and the experimental area (D). E shows the direction of the projection and camcorder. The tank was lined and divided with opaque black corrugated plastic except for the front side of the tank which was lined with a translucent film and the dividers between the companion compartments and the refuge where 2mm plastic mesh was used to allow visual and olfactory contact between the companion and test fish (Ioannou *et al*. 2012).

**Fig S2.** The probability of an attack being made further from the centroid than the mean prey distance against time taken to leave the refuge for different prey densities (black to light grey indicates least dense to most dense prey groups).

**Fig S3**. The probability of an attack occurring closest to the refuge as a function of the time taken to leave the refuge across different densities (black to light grey indicates least dense to most dense prey groups).
