# Supplementary figures and images for: Marginal predation: do encounter or confusion effects explain the targeting of prey group edges?
Source: Behav Ecol. 2017 Jul 27;28(5):1283–92. doi: 10.1093/beheco/arx090 (PMC5873256; doi:10.1093/beheco/arx090)

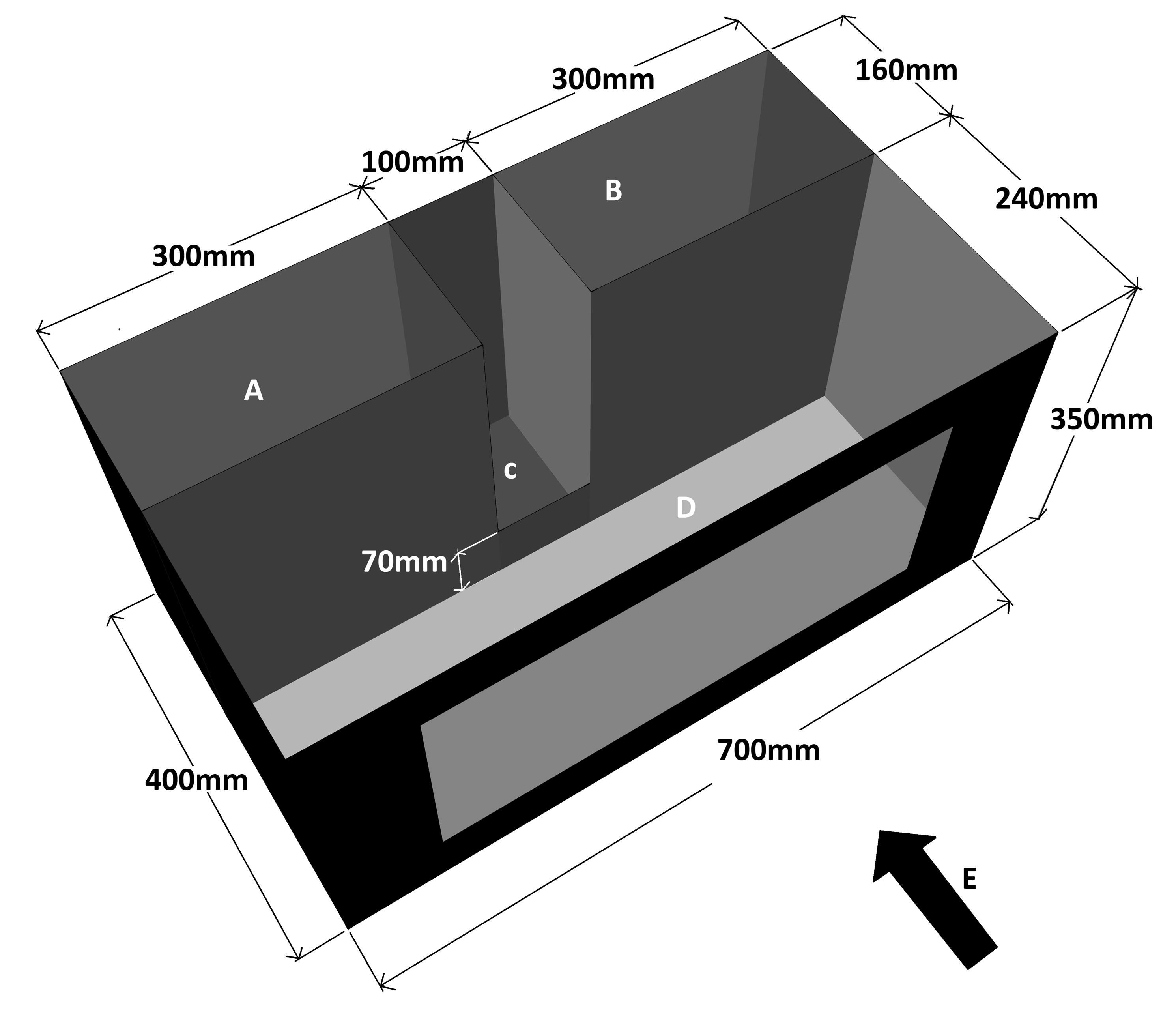

Supplement: Duffield Figure S1 [file arx090_suppl_duffield_figure_s1.png]

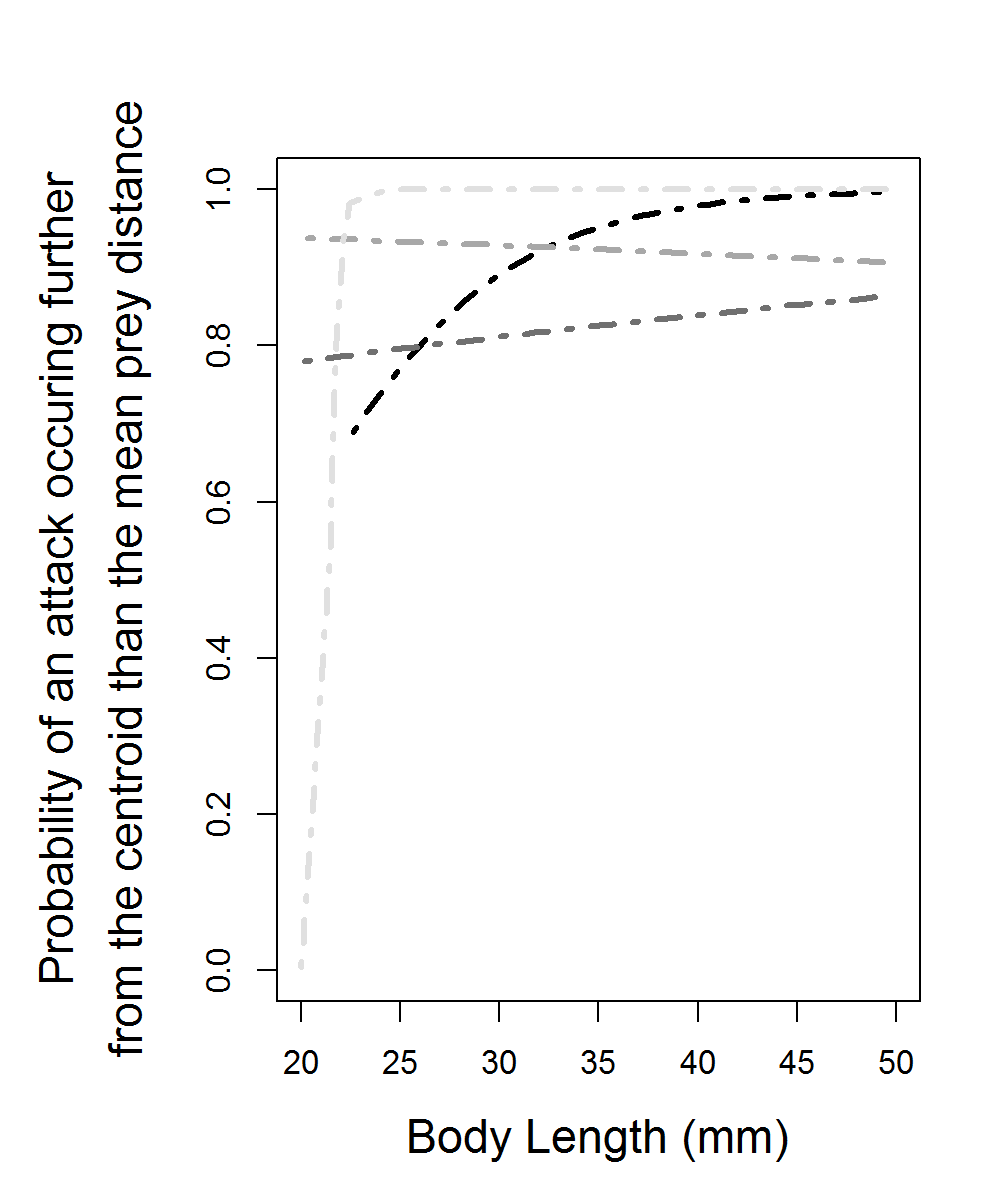

Supplement: Duffield Figure S2 [file arx090_suppl_duffield_figure_s2.png]

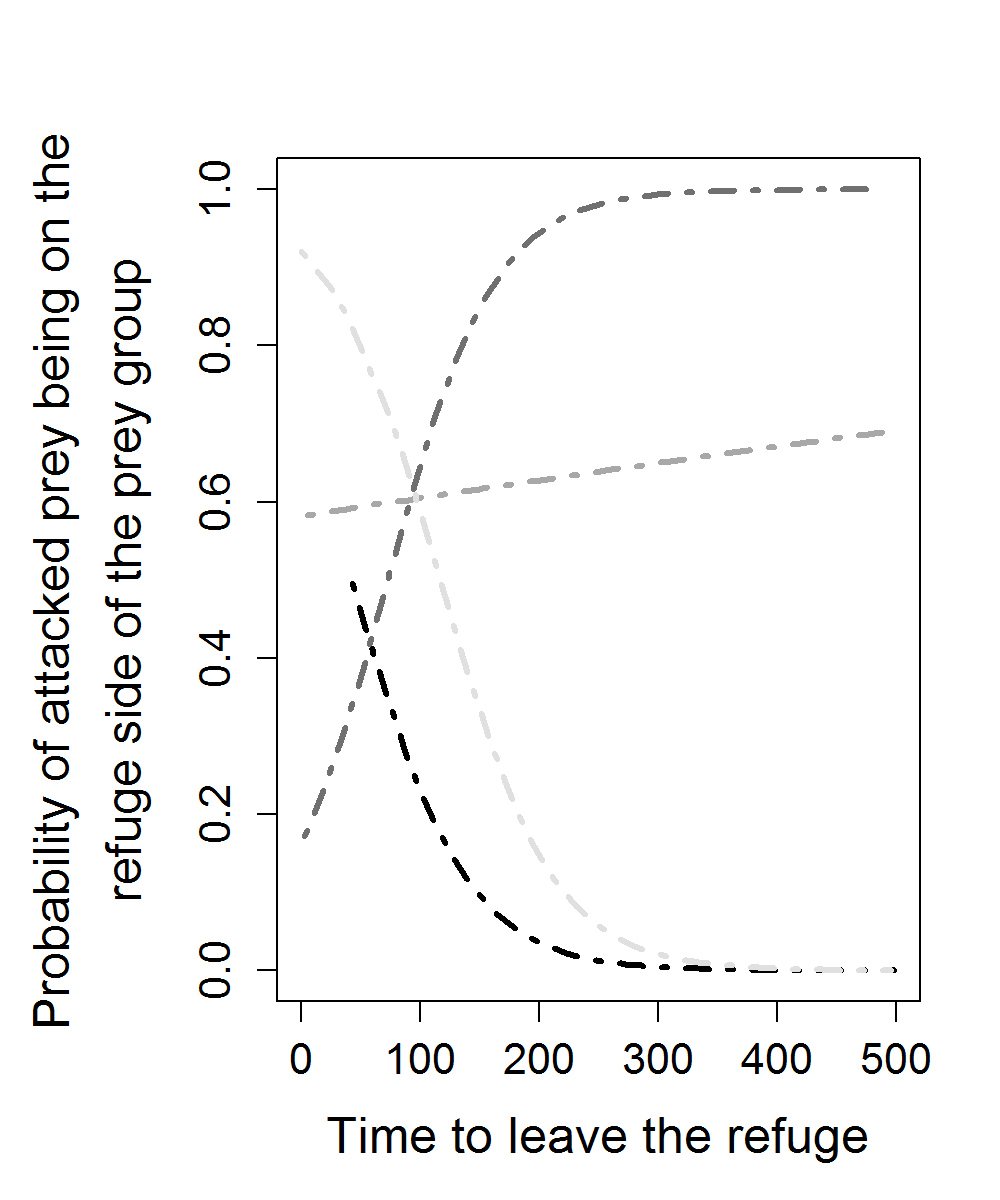

Supplement: Duffield Figure S3 [file arx090_suppl_duffield_figure_s3.png]
